# ALTERNATIVE PATHS TO AN AGE-FRIENDLY COMMUNITY

**DOI:** 10.1093/geroni/igad104.1509

**Published:** 2023-12-21

**Authors:** Shayna Gleason, Patricia Oh, Caitlin Coyle, Ceara Somerville

**Affiliations:** University of Massachusetts, Boston Gerontology, Boston, Massachusetts, United States; UMaine Center on Aging, Bangor, Maine, United States; University of Massachusetts Boston, Boston, Massachusetts, United States; University of Massachusetts Boston, Boston, Massachusetts, United States

## Abstract

While the age-friendly community movement has seen tremendous progress in the past decade, with over 700 U.S. member communities in the Network of Age-Friendly States and Communities (NAFSC), many more U.S. communities have not yet joined the network. Joining NAFSC can spur activity to improve local conditions for older residents, as well as allow communities access to informational resources and contacts from peer communities that have undertaken similar initiatives. A better understanding of the barriers preventing communities from joining NAFSC, the hesitancy about joining, and the age-friendly work being done outside of NAFSC is critical to extending age-friendly work nationally. The present study explored factors that inhibit communities from joining NAFSC. We conducted 12 semi-structured virtual interviews with community leaders in Maine and Massachusetts whose communities are not NAFSC members but are actively working on aging-related issues. Participants were recruited through the researchers’ professional networks. We intentionally cultivated diversity of community size during recruitment. Transcripts and interview notes were coded thematically. Resulting themes included a lack of knowledge/misconceptions about what joining NAFSC requires, a perception that there are few benefits to joining, political concerns, insufficient personnel capacity, other financial priorities (e.g., children’s programming, COVID relief), and a desire to retain local control over aging-related initiatives. Recommendations include clearer communication of NAFSC membership requirements and benefits, additional technical assistance to communities, and loosening of reporting requirements for small communities.

